# Draft Genome Sequences of 10 Clinical K2-Type Klebsiella pneumoniae Strains Isolated in Russia

**DOI:** 10.1128/MRA.01023-18

**Published:** 2018-10-11

**Authors:** Nikolay V. Volozhantsev, Angelina A. Kislichkina, Anastasia I. Lev, Ekaterina V. Solovieva, Vera P. Myakinina, Tatiana N. Mukhina, Alexandr G. Bogun, Nadezhda K. Fursova

**Affiliations:** a State Research Center for Applied Microbiology and Biotechnology, Obolensk, Moscow, Russia; University of Delaware

## Abstract

We report here the genome sequences of 10 Klebsiella pneumoniae strains of capsular type K2 isolated in Russia from patients in an infectious clinical hospital and neurosurgical intensive care unit. The draft genome sizes range from 5.34 to 5.87 Mb and include 5,448 to 6,137 protein-coding sequences.

## ANNOUNCEMENT

Klebsiella pneumoniae is a common cause of community- and hospital-acquired infections, especially in immunocompromised patients (1, 2). Of the more than 80 capsular serotypes, K. pneumoniae strains of the K2 type, along with K1, are the most virulent human pathogens (3, 4). Recently, these features were confirmed for the strains isolated from the patients of an infectious clinical hospital and a neurosurgery intensive care unit in Russia (5).

Here, we report the genome sequences of 10 K. pneumoniae strains of capsular type K2 isolated from patients in an infectious clinical hospital (Table 1, KPi strains) and neurosurgical intensive care unit (Table 1, KPB strains).

**TABLE 1:** Strain-identifying information and basic statistics on assemblies and annotations

Strain name	Isolation source	SCPM ID^*a*^	SRA accession no.	GenBank accession no.	GC content (%)	Genome size (bp)	No. of contigs	No. of genes	Plasmid replicon type(s)	Cluster
KPB755	Urine	SCPM-O-B-8392	SRR7152495	QIUF00000000	56.7	5,871,891	163	6,137	IncR, IncHI1B, IncL/M, IncFIB	I
KPi4341	Sputum	SCPM-O-B-8394	SRR7152498	QIUE00000000	57.1	5,582,582	179	5,845	IncR	I
KPi3310	Urine	SCPM-O-B-8393	SRR7152497	QIUD00000000	57.1	5,547,691	119	5,801	IncR, ColRNAI, IncL/M	I
KPB1299	Urine	SCPM-O-B-8395	SRR7152499	QIUB00000000	57.3	5,340,960	76	5,478	IncR	I
KPi6208	Trachea	SCPM-O-B-7666	SRR7152494	QIUI00000000	57.2	5,432,259	96	5,531	IncHI1B	II
KPB492-16	Purulent wound	SCPM-O-B-8390	SRR7152501	QIUJ00000000	57.3	5,423,933	72	5,448	IncHI1B	II
KPi2965	Sputum	SCPM-O-B-7936	SRR7152496	QIUG00000000	57.2	5,448,378	126	5,555	IncHI1B, IncA/C2	II
KPB463-16	Bronchial lavage fluid	SCPM-O-B-8391	SRR7152493	QIUH00000000	57.2	5,412,386	121	5,489	IncHI1B	II
KPi1748	Trachea	SCPM-O-B-8040	SRR7152502	QIUK00000000	56.9	5,558,241	81	5,597	IncHI1B	III
KPi3014	Sputum	SCPM-O-B-7851	SRR7152492	QIUL00000000	56.8	5,777,652	158	6,039	IncHI1B, IncQ1, IncFIB	IV

a SCPM, State Collection of Pathogenic Microorganisms “Obolensk”; ID, identifier.

Bacterial cultures were grown at 37°C on nutrient medium no. 1 (SRCAMB, Obolensk, Russia). Genomic DNA was extracted using the standard phenol-chloroform extraction and ethanol precipitation methods. Whole-genome sequencing was performed using an Illumina MiSeq instrument according to the manufacturer’s instruction. The Nextera DNA library preparation kit and MiSeq version 3 (300 cycles) reagent kit were used for sequencing, as previously reported (6). Reads without quality filtering were *de novo* assembled using SPAdes 3.9.0 with default parameters (7). The assembled genomes were annotated using the NCBI Prokaryotic Genome Annotation Pipeline (8). Finally, we obtained from 72 to 179 contigs for each genome (Table 1). The draft genome sizes ranged from 5.34 to 5.87 Mb, with the GC content ranging from 56.7 to 57.3%.

The sequences of seven plasmid replicon types (IncHI1B, IncR, IncL/M, IncFIB, ColRNAI, IncA/C2, and IncQ1) were identified in the genomes by using PlasmidFinder (9). Antibiotic resistance and virulence genes were identified using ResFinder version 2.1 (10) and VirulenceFinder version 1.2 (11), respectively.

The strains were distributed into four clusters based on the bacterial core genome single nucleotide polymorphism (SNP) analysis with Wombac 2.0 (VBC, Monash University, Australia). Comparative genomic analysis revealed important differences in antibiotic resistance and virulence genes between strains of different clusters. The strains of cluster I (KPB755, KPi4341, KPi3310, and KPB1299) were multidrug resistant. In the genomes of these strains, we found genes determining resistance to beta-lactams (*bla*_SHV-11_, *bla*_TEM-1B_, *bla*_CTX-M-15_, *bla*_OXA-1_, and *bla*_OXA-48_), aminoglycosides [*aac(6′)Ib-cr* and *aac(3)-IIa*], fluoroquinolones (*qnrS1*, *oqxA,* and *oqxB*), phenicols (*catA1* and *catB4*), tetracycline (*tetA*), fosfomycin (*fosA*), trimethoprim (*dfrA1*), and sulfonamides (*sul1*). On the contrary, strains of clusters II and III (KPi6208, KPB492-16, KPi2965, KPB463-16, and KPi1748) did not contain a wide arsenal of resistance genes but carried genes specific for hypervirulent (hvKP) K. pneumoniae (2) as follows: *rpmA* and *rmpA2* , genes coding for regulators of the mucoid phenotype; *wabG* and *uge*, genes involved in the synthesis of the polysaccharide capsule and lipopolysaccharide; and the *iroBCDN* operon coding for the iron transport system. Moreover, the strains of clusters II and III possessed a hypermucoviscous phenotype identified by the string test (2). The strain of cluster IV (KPi3014) contains both a large set of antibiotic resistance genes [*bla*_TEM-1B_, *aph(6)-Id*, *aph(3′)-Ia*, *aph(3″)-Ib*, *oqxA, oqxB*, *fosA*, *catA1, sul2, tetA*, and *dfrA1*] and a set of virulence genes characteristic of hvKP strains, with the exception of the *iroN* gene of the *iroBCDN* operon.

The presented genome sequences can facilitate an understanding of the genetic diversity within K. pneumoniae clinical isolates.

### Data availability.

These genome sequences were deposited in GenBank/ENA/DDBJ and the SRA under the accession numbers listed in Table 1.
